# An exploratory study of pelvis anatomy to revise the bony canal used for LC2 screw insertion

**DOI:** 10.1186/s12891-022-05256-2

**Published:** 2022-03-26

**Authors:** Jialiang Guo, Weichong Dong, Zhiwei Zhang, Ruipeng Zhang, Yingchao Yin, Wei Chen, Yingze Zhang, Zhiyong Hou

**Affiliations:** 1 The School of Medicine, Nankai University, Tianjin, P.R. China; 2 Department of Orthopaedics, The Third Hospital of Hebei Medical University, Shijiazhuang, P.R. China; 3 Department of Pharmacy, The Second Hospital of Hebei Medical University, Shijiazhuang, P.R. China; 4 Department of Anesthesiology, The People’s Hospital of Hongan, Huanggang, P.R. China; 5 NHC Key Laboratory of Intelligent Orthopeadic Equipment (The Third Hospital of Hebei Medical University), Shijiazhuang, P.R. China; 6 Chinese Academy of Engineering, Beijing, P.R. China

**Keywords:** Pelvis anatomy, Pelvic fracture, LC2 screw, Bony canal

## Abstract

**Background:**

Percutaneous screw placement, especially the insertion of LC2 screws, is technically demanding. Although the traditional LC2 bony canal spans the anterior inferior iliac spine (AIIS) to the posterior superior iliac spines (PSIS), a high perforation rate has been reported.

**Objection:**

The aim of this research was to design a revised bony canal, measure the canal width and length and guide the insertion of LC2 screws for pelvic fractures.

**Materials and methods:**

The plane tool in the Mimics analysis menu was used to draw a midplane connecting the midpoint between the anterior inferior spine and the PSIS upper flat region with pelvic CT data. The minimum widths of the upper, middle, lower surfaces of the tunnel and perforation rate were measured and compared. The ideal screw length was also measured along the longitudinal axis running through the midpoint of the midplane.

**Results:**

The minimum widths of the upper, middle and lower surfaces of the revised canal were 3.63 mm, 7.7 mm, and 11.93 mm, respectively, in males and 5.97 mm, 9.93 mm, and 12.45 mm, respectively, in females. Significant differences were observed among the upper, middle and lower surfaces of the revised canal in male patients (*P* < 0.001). In female subjects, the upper canal surface was significantly different from the middle and lower canal surfaces (P < 0.001). The perforation rate was significantly decreased especially in females pelvic. The channel length passing through the midpoint of the narrowest position of the pelvis was 130.85 ± 8.02 mm in males and 124.30 ± 7.71 mm in females and was significantly different for male and female pelvises (*P* = 0.004).

**Conclusion:**

The LC2 screw should be inserted along the intersection line of the AIIS lateral wall and the iliac body. The screw should be inserted under the line between the midpoint of the AIIS and the PSIS upper flat region to ensure accuracy of placement. LC2 screws can be more easily inserted in males than in females, and the rate of cortical perforation can be significantly decreased under the guidance of the newly proposed canal.

## Introduction

Pelvic and acetabular fracture treatment has always been challenging for orthopedic surgeons. To treat iliac crescent fractures or sacroiliac fracture dislocations, anterior or posterior open reduction internal fixation is always advocated, although there are no clear reports of the rate of perioperative infection or other complications [1]. As more in-depth research on the pelvis has been performed, minimally invasive techniques (percutaneous) have become a trend and have good clinical outcomes for the treatment of specific types of pelvic fractures [2–4]. However, the safety and accuracy of screw insertion have always been problematic, and long-term exposure to radiation may affect health care teams or patients because of prolonged fluoroscopy times.

Berry analyzed two intrailiac anchor paths [5]. The easiest intrailiac path is one in which the canal lies between the posterior superior iliac spines (PSIS) just above the sciatic notch and intersects with the top of the acetabulum: the diameter of this passage ranges from 14.3 mm to 27.3 mm, but the passage limits the anchorage length because of the risk of acetabular involvement. To reduce relative complications, cannulated “LC2 screws” were inserted through the supraacetabular tunnel to treat iliac crescent fractures in 2002 [6]. LC2 screw insertion or the Infix technique can be used to preserve blood supply to the bone and reduce blood loss and surgical site infection rates compared with open internal fixation [6–8].

However, percutaneous screw placement is technically demanding, and the insertion of guide wires for LC2 screws along the supraacetabular tunnel can sometimes be challenging, because standard instruments may interfere with the C-arm in the “Teepee” view. Berry reported that the narrowest point along this canal is always located 60–65 mm from the entry of the intrailiac path at the level of the sacrum [5]. Therefore, radiographic research is required in conjunction with a relative anatomical study to guarantee effective utilization of LC2 screws. Although the traditional LC2 bony canal spans the anterior inferior iliac spine (AIIS) to the PSIS, a high perforation rate (43%) has been reported [9]. The aim of this research study was to design a revised bony canal, measure the canal width, length, compare perforation rate, and guide the insertion of LC2 screws for pelvic fractures.

## Materials and methods

In this retrospective study, pelvic CT data collected from 2016.2–2019.8 were evaluated in an orthopedics study. Ethical approval was obtained from the Regional Ethics Committee of our hospital, and the study was conducted in accordance with the guidelines provided by the Declaration of Helsinki. Informed signed consent was obtained from all the patients who enrolled in the study. The inclusion criteria were that the patients were older than 18 years with complete initial CT imaging data. The exclusion criteria were patients with CTs with B70 or slices larger than 2 mm. The subjects were divided into groups of different sexes based on age: Group I for males (77 men, 47.7 ± 14.9 years) and Group II for females (24 women, 51.6 ± 14.8 years).

### The bony tunnel construction for LC2 screws

Data on normal semipelvises were collected for 77 men and 24 women (with an average age of 48.6 ± 14.9 years). The CT data were obtained from PACS in our hospital and were subsequently reconstructed to design a revised bony tunnel for the insertion of LC2 screws. The data were measured with Mimics 21.0 (Materialise, Leuven, Belgium), and the same Hounsfield units of 226 (minimum) and 1600 (maximum) were identified as the threshold for the bone tissue in all patients. Then, the bone segments and construction functions in Mimics were used to obtain three-dimensional images of the tunnels.

**Fig. 1:**
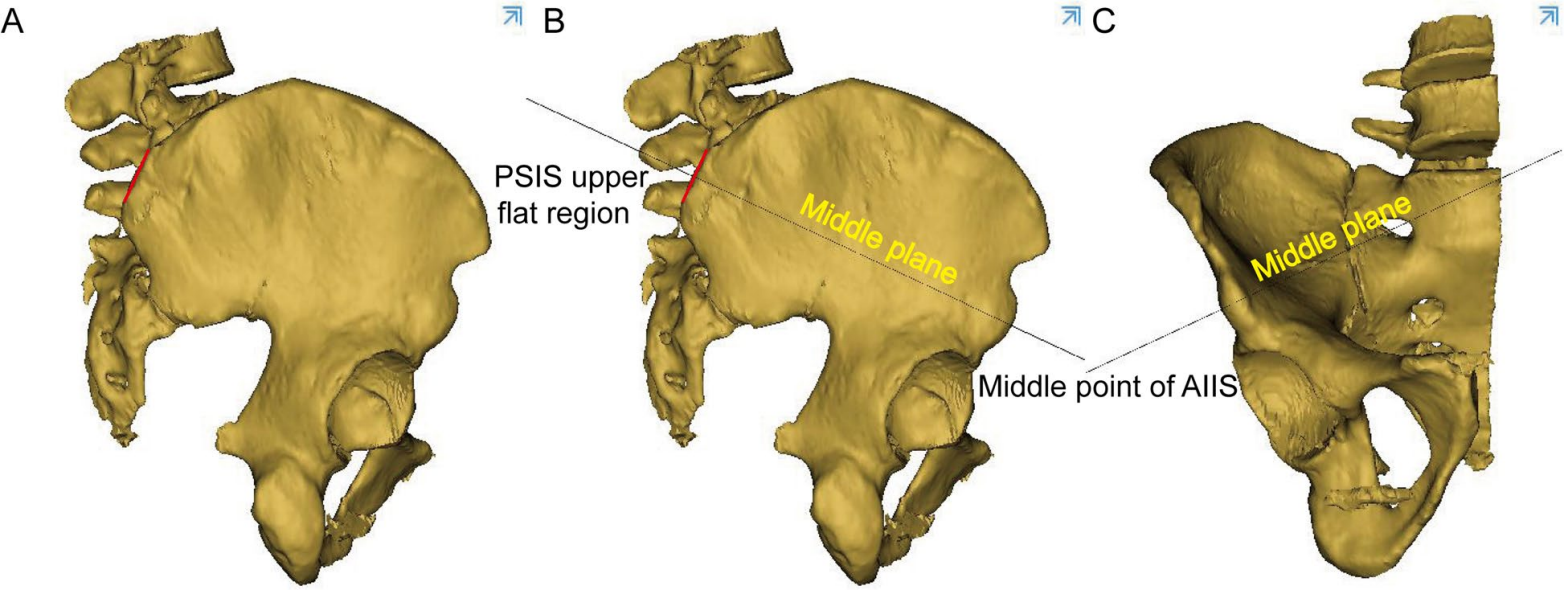
The midplane was identified. **A** 3D construction image for semipelvic. **B** The middle plane in lateral view. **C** The middle plane in AP view

**Fig. 2:**
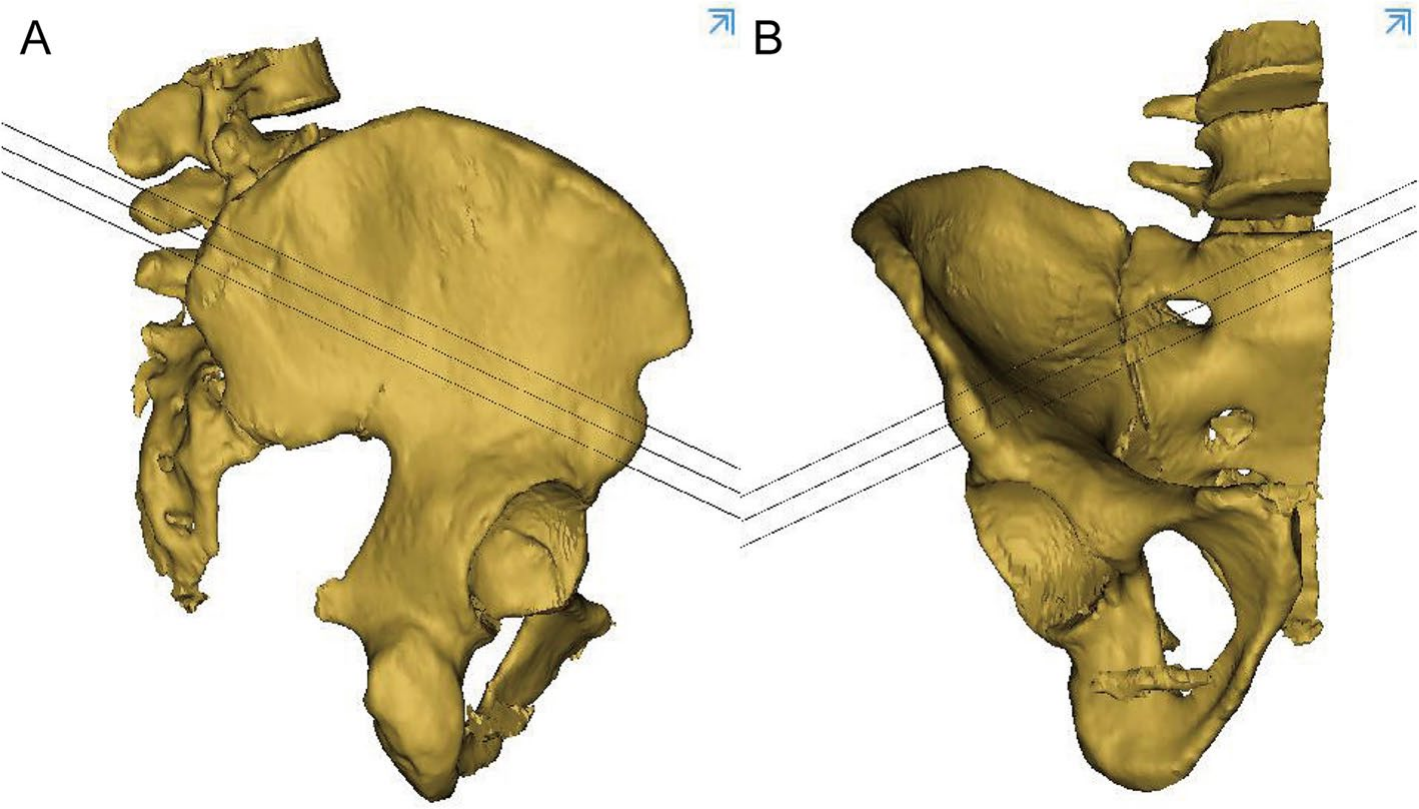
The boundary of the identified bony tunnel was formed by two other planes were drawn parallel to the midplane

**Fig. 3:**
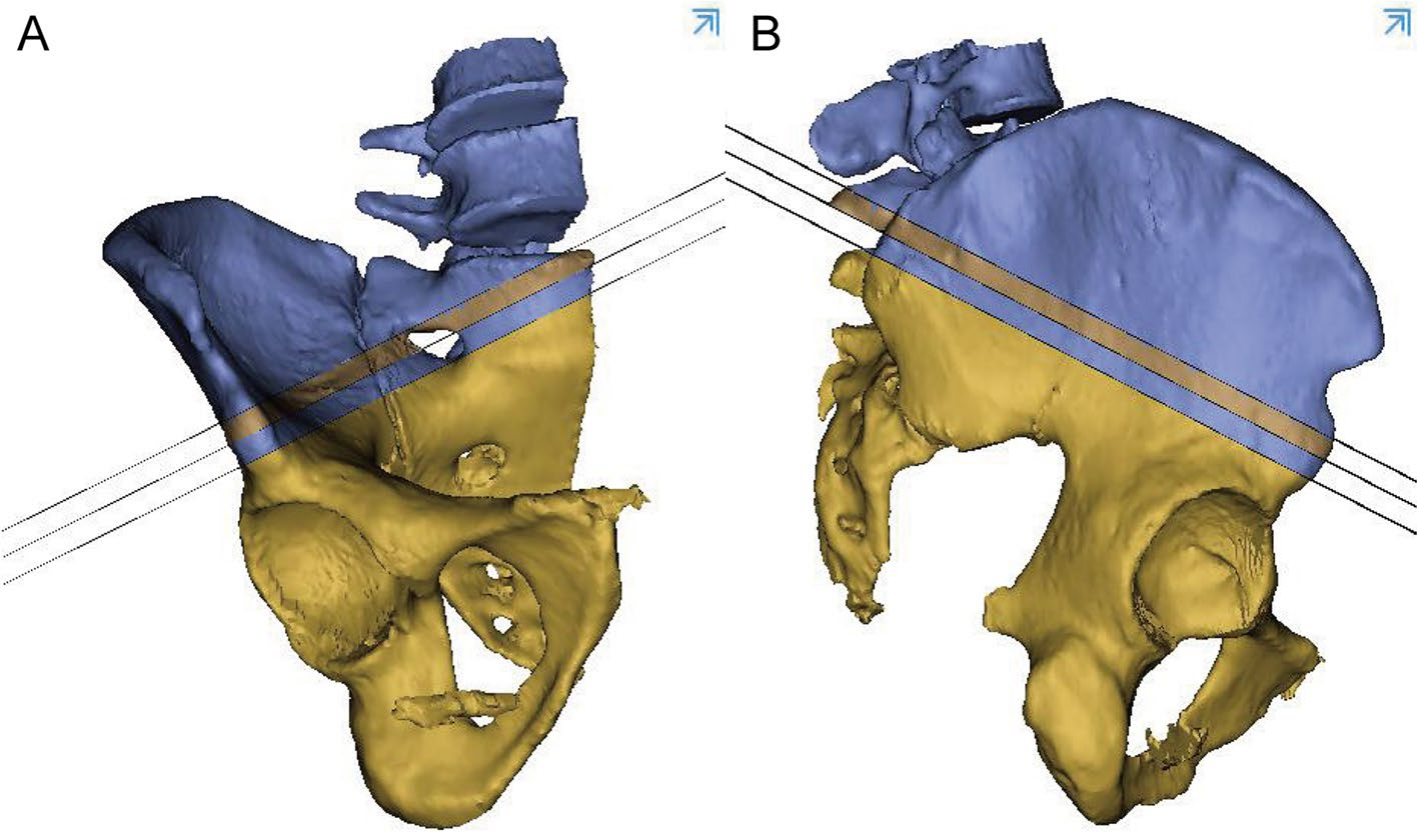
The revised bony channel within 6.5 mm (13 mm) above and below the midplane

**Fig. 4:**
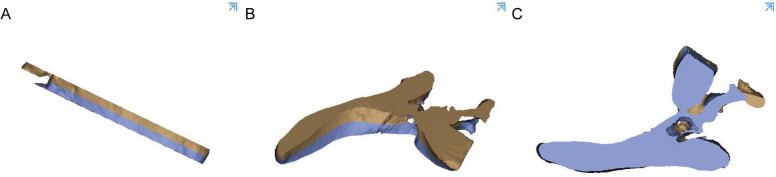
The tunnel for the insertion of the LC2 screws was identified with colorful images

The 3D pelvis images were analyzed using the Mimics medical workstation. The plane tool in the analysis menu was used to draw a cutting plane using three points: the anterior and posterior points at the midpoint of the anterior inferior spine and the midpoint of the PSIS upper flat region, and was names as midplane (Fig. 1). The PSIS upper flat region was marked in the figure and was characterized by a broad bottom surface and a distinctly different curvature (almost 0) from that of other parts of the iliac region (Fig. 1A).Two other planes were drawn parallel to the midplane to form the boundary of the identified bony tunnel (Fig. 2). The tunnel for the insertion of the LC2 screws was identified as the revised bony channel within 6.5 mm (13 mm) above and below the midplane (Figs. 3 and 4).

### Measurement of parameters of the upper, middle and lower surfaces of the bony canal

**Fig. 5:**
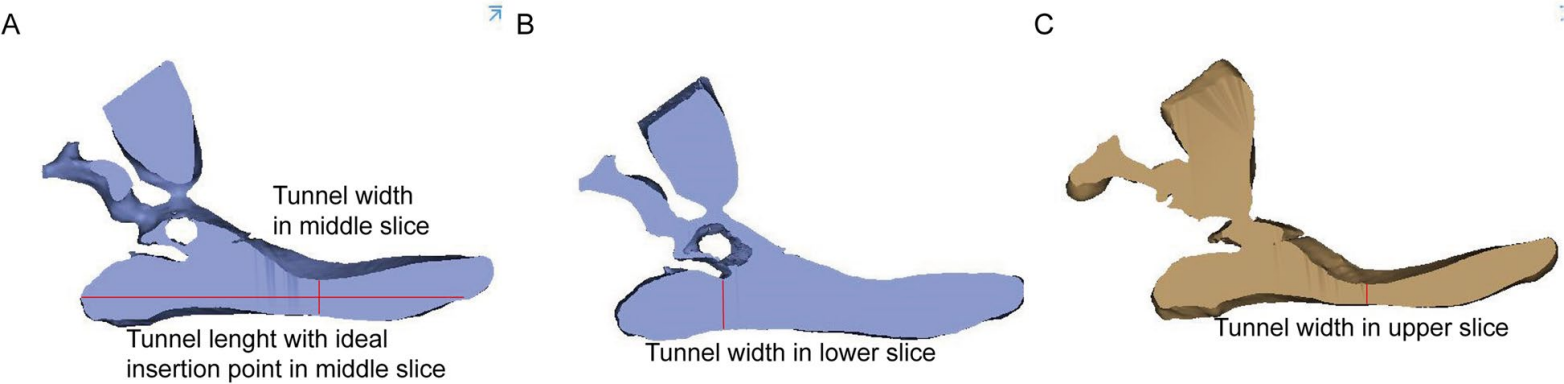
The ideal tunnel length and the narrowest width was calculated. **A** The ideal tunnel length was identified as the maximum distance between the insertion point of the LC2 screw and the PSIS upper flat region. **B** The tunnel width in lower surface. **C** The tunnel width in upper surface

“Measure distance” tools were used to calculate the ideal length and minimum width of the bony tunnel. The ideal tunnel length was identified as the maximum distance between the insertion point of the LC2 screw and the PSIS upper flat region (Fig. 5). The insertion point of the LC2 screw was located at the lateral margin of the midpoint of the anterior inferior iliac spine, avoiding the attachment point of the hip capsule. The minimum tunnel width consisted of the minimum width of the upper surface, the width of the midplane and the width of the lower plane.

**Fig. 6:**
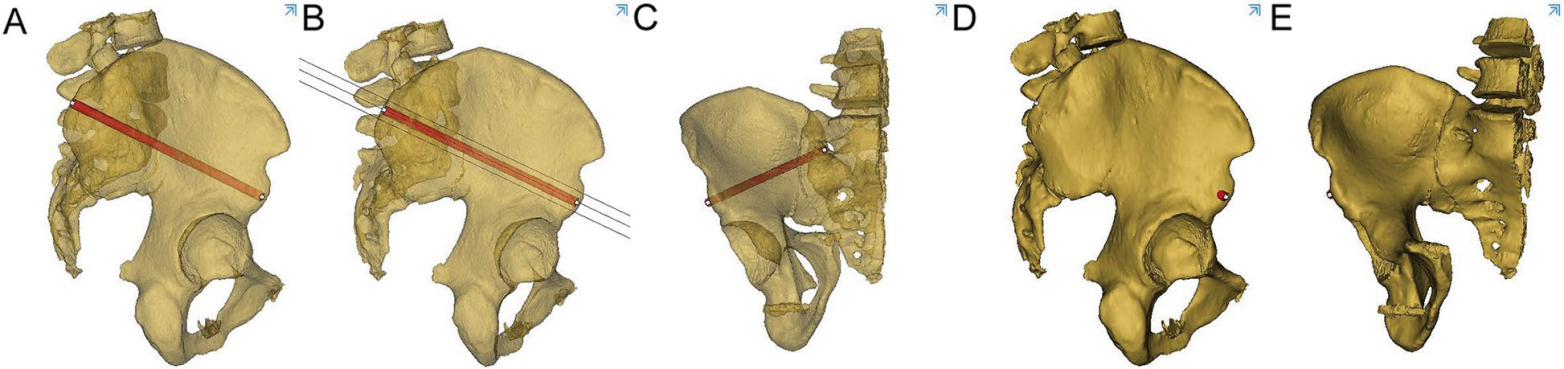
The transparence tool in Mimics was used to create a translucent illustration of the ideal LC2 screw tunnel

**Fig. 7:**
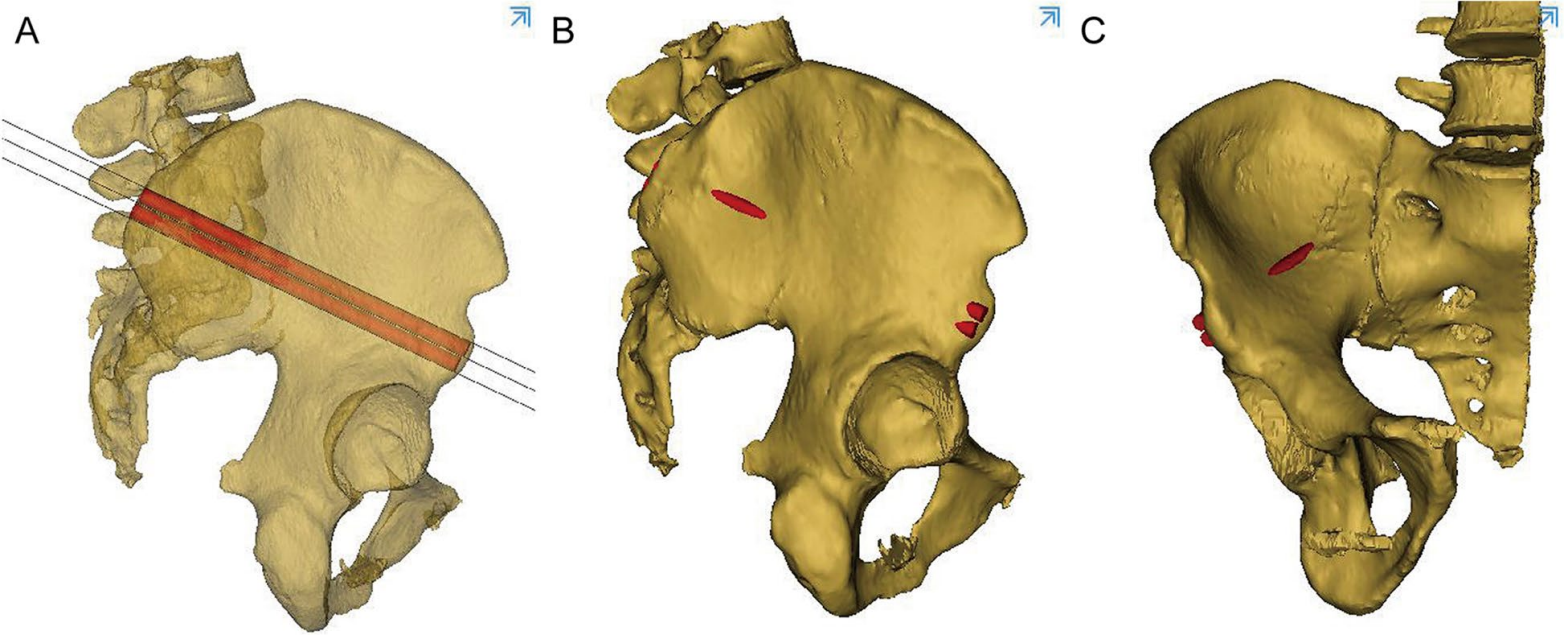
Two simulated screws were inserted into the space among the three cutting planes, and the cortical perforation of the screws was visualized using 3D reconstruction images

A cylinder with a diameter of 6.5 mm was drawn to simulate the LC2 screw at the ideal insertion point. Three parallel cutting planes were located 6.5 mm equidistant from each other, and the ideal insertion tunnel lay along the longitudinal axis running through the midpoint of the midplane. The transparence tool in Mimics was used to create a translucent illustration of the ideal LC2 screw tunnel (Fig. 6). Two simulated screws were inserted into the space among the three cutting planes, and the cortical perforation of the screws was visualized using 3D reconstruction images (Fig. 7).

### Statistical analysis

Continuous data were presented as a mean and standard deviation. The homogeneity of variance for the continuous variables was evaluated by the Levene test. Comparisons between male and females groups were performed by Mann-Whitney U test or χ^2^ to determine the differences. Spearman correlations were calculated, and the linear or nonlinear correlation coefficients were used to assess the correlation between age and the measurement results. The following criteria were used for the correlation parameters. Values of |r| ≥ 0.8 corresponded to a high correlation between two variables. Values of 0.5 ≤ |r| < 0.8 corresponded to a moderate correlation between two variables. Values of 0.3 ≤ |r| < 0.5 corresponded to a low correlation between two variables. Values of |r| < 0.3 corresponded to no correlation between two variables.

## Results

**Fig. 8:**
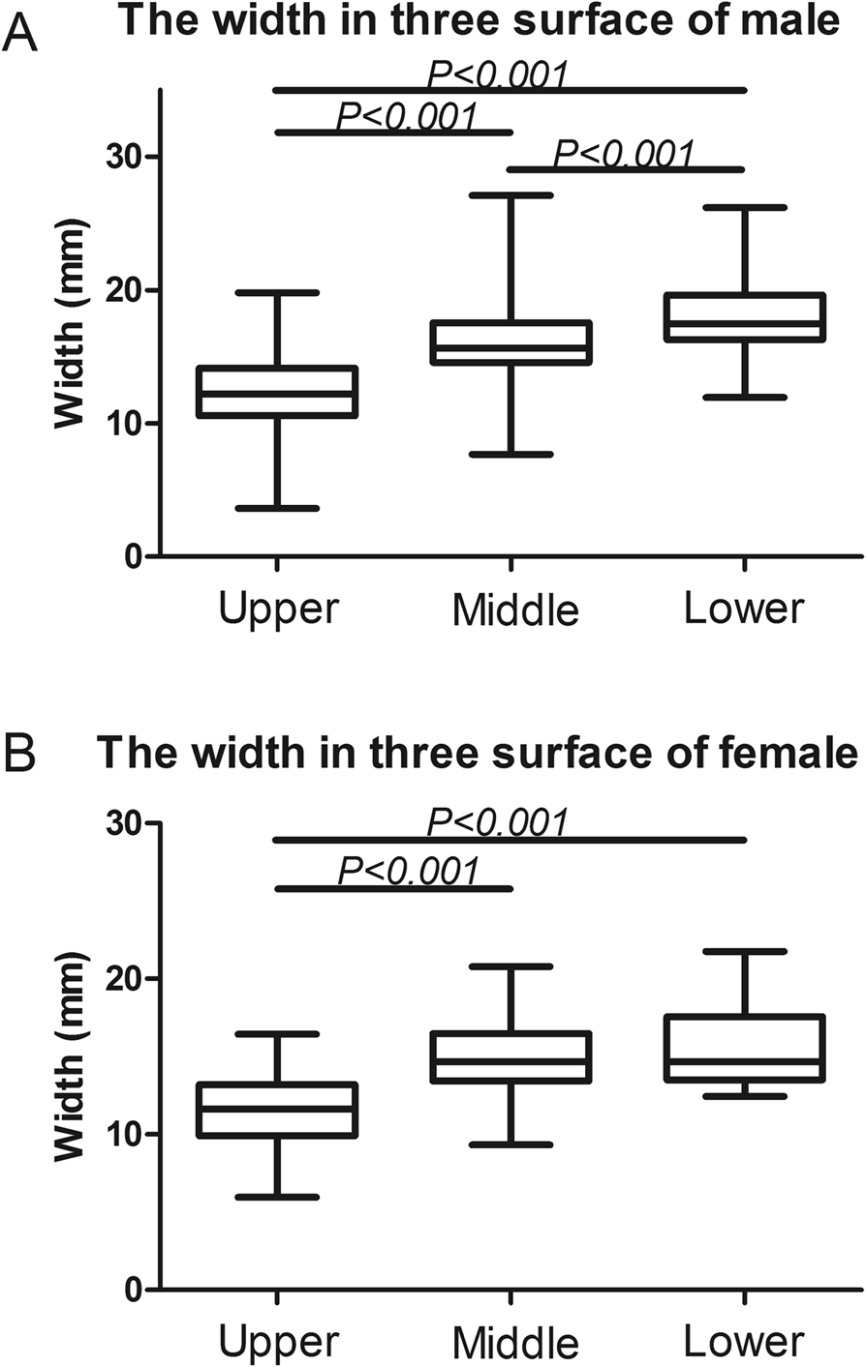
The width in three surface in man and woman respectively

A total of 101 patients (77 males vs. 24 females) with normal semipelvises were included in this study (the average age of the patients was 48.6 ± 14.9 years). The width for screw insertion increased when the cutting planes approached the acetabulum. Significant differences were observed among the upper, middle and lower surfaces of the revised canal in male patients (*P < 0.001*). In female subjects, the upper surface of the revised canal was observed to be significantly different from the middle and lower surfaces (*P < 0.001*) (Fig. 8).

There was no significant decrease in the minimum width for screw insertion for the canal upper surface between male (12.12 ± 2.89 mm) and female (11. 20 ± 2.71 mm; *P* = 0.165) pelvic tissue. The minimum width for screw insertion was 3.63 mm in males, and 5.97 mm in females (Table 1). The narrowest region of the canal upper surface was found to lie almost within the iliac fossa of the hip bone.

**Table 1:** The minimal width in three surface in male and female respectively

Sex	Upper (mm)	Middle (mm)	Lower (mm)	Channel length
Male	12.12 ± 2.89	16.22 ± 2.95	18.07 ± 2.76	130.85 ± 8.02
Female	11. 20 ± 2.71	14.97 ± 2.81	15.65 ± 2.49	124.30 ± 7.71
Z	−1.388	− 1.660	− 3.487	−2.912
*P*	0.165	0.097	< 0.001*	0.004*

*means that there was significant differences

There was also no significant differences in the width of the pelvic midplane between males and females (16.22 ± 2.95 mm vs. 14.97 ± 2.81 mm; *P* = 0.097). The minimum pelvis midplane width was 7.7 mm in males and 9.93 in females. The minimum pelvic width at the lower canal surface was significantly lower for females compared to males (15.65 ± 2.49 mm vs. 18.07 ± 2.76 mm; *P* < 0.001) (Table 1). The minimum pelvic width was 11.93 mm in males and 12.45 mm in females. The narrowest region on the lower canal surface lay almost within the posterior region of the sacroiliac joints. There was no correlation between these 3 parameters and age.

The channel length passing through the midpoint of the narrowest region was 130.85 ± 8.02 mm in males and 124.30 ± 7.71 mm in females and was significantly different for male and female pelvises (*P* = 0.004). The LC2 screw was simulated in 3-Matic and easily inserted along the midplane (Table 1). However, LC2 screws inserted above the midplane were more prone to cortical perforation than those inserted along or below the midplane. The female had a significant high rate of perforation when the screw was inserted above the midplane compared with insertion along or below the midplane, or compared with males above the midplane (Table 2). The LC2 screw length was also not correlated with age.

**Table 2:** Cortical bone perforation with different insertion canal in males and females

Position relative to midplane	Above (%)		Along (%)		Below (%)		Pearson χ^2^	*P*
Perforation or not	In	Out	In	Out	In	Out		
Male	58 (75.3)	19 (24.7)	63 (81.8)	14 (18.2)	69 (89.6)	8 (10.4)	5.397	0.067
Female	13 (54.2)	11 (45.8)	16 (66.7)	8 (33.3)	22 (91.7)	2 (8.3)	8.471	0.014*
Pearson χ^2^		3.923		2.465		0.087		
		0.048*		0.115		0.768		

*means that there was significant differences

## Discussion

This study focused on the morphological channel used for LC2 screw insertion. LC2 screws constitute an important surgical strategy to treat LC2 iliac crescents or acetabular anterior column fractures, but a high perforation rate has been reported using the traditional canal [9]. In this study, a different plane from the traditional canal was chosen to reduce the rate of LC2 perforation. The results of this study led to the conclusion that LC2 screws should be inserted below the connecting plane of the midpoint of the AIIS and PSIS upper flat regions. Perforation occurred easily for LC2 screw insertion above the identified midplane. The morphological characteristics of the revised LC2 screw channel were determined in this study, and it was concluded that the tip of the LC2 screw should be inserted at the midpoint between the PSIS upper flat region and AIIS. The results of this research study can help ensure the successful use of LC2 screws in treating pelvic fractures.

Percutaneous screw fixation of iliac wing fractures is an established technique but remains technically demanding. It has been reported that misplaced screws produce many complications, such as injuries to the surrounding soft tissue and proliferation of vessels or nerves. In traditional treatment for lateral compression fractures, radiographic imaging must be performed and several positions of the C-arm must be considered before operation to plan the exact positioning of LC2 screws. Currently, a “Teepee” view along the supraacetabular bony canal can provide an optimal view for guide-wire insertion. However, obtaining a Teepee view several times and adjusting the guide pin may increase radiographic exposure and surgical time. Therefore, an auxiliary method, such as anatomical measurement of the bony canal parameters, is needed to support LC2 screw insertion. Therefore, considering the high rate of LC2 screw perforation using the traditional method, a revised midplane was proposed in this study to evaluate the anatomic characteristics of the LC2 bony canal. The plane between the AIIS and middle PSIS upper flat region was chosen because the PSIS is not obvious in all patients, and the bottom surface of the chosen plane is wide and flat. Furthermore, the PSIS upper flat region can more easily observed intraoperatively by radiography than the PSIS.

When the LC2 screw was first introduced, Starr reported that the illum became thicker near the greater sciatic notch, but the precise parameters of the bony canal were not obtained [6]. Berry measured the length extending from the widest point of the posterior superior iliac spine (PSIS) to the tip of the anterior inferior iliac spine (AIIS) using specific points in specimens and reported that the corresponding bony canal provided a longer and potentially safer anchoring site than that identified by Starr [5]. Due to technical limitations, the internal bony canal was only measured using normal CT, which can only be used to perform measurements along one cutting direction [10]. Mimics enables precise measurement of the bony canal identified by Berry or a revised version. Mimics can visualize internal structure for cutting the pelvis along any designed direction. After defining the direction of the optimal osseous channel for the LC2 screw, the revised bony canal for the screw was comprehensively measured. Two aspects should be considered for LC2 screw insertion: the minimum width of the osseous channel cross section, which determines the screw diameter, and the screw length for ideal insertion.

In this study, the narrowest widths of three surfaces of the bony canal were measured to determine the optimal position for LC2 screw insertion. The width for screw insertion was found to increase for cutting planes approaching the lower surface and decrease for cutting planes approaching the upper surface. McCord studied 10 different fixation techniques and found that the longer the iliac nail in the iliac crest was, the more stable the nail was [11]. However, the irregularity of the LC2 bony channel results a different length for the inserted screw than the maximum length of the bony channel. Furthermore, the narrowest canal inner diameter should not be simply taken as the maximum diameter of the inserted LC 2 screw, because the central axis of the screw channel does not overlap with that of the narrowest channel. To determine the optimal insertion position, a simulation was performed for screw placement in the midplane and among the three planes of the revised LC2 screw tunnel. The length of the screw inserted through the midpoint of the narrowest region was selected as the optimal screw length, and this midpoint intersected with the central axis of the screw channel. The insertion point of the screw was also effectively determined through simulation, and the intersection of the midpoint of the AIIS and the iliac body was determined to be the ideal insertion point of the LC2 screw. Therefore, it was concluded that it was safe to insert screws along or below the middle cutting plane, where the screw lengths were comparable to those employed using the traditional method.

Limitations were placed on the number of enrolled subjects, and more patients will be included in future studies. The traditional canal lying just under the revised canal was not measured but reported in previous studies, and the minimal width of the traditional canal was 12.3 ± 2.6 mm and 9.2 ± 3.0 mm in male and female, which was comparable with our revised canal [10]. Therefore, it further confirmed the research value of the revised bony canal proposed in this study. In our next study, more anatomic parameters will be calculated to assist with LC2 screw insertion, and boundaries for LC2 screws will be accurately identified.

## Conclusion

An LC2 screw should be inserted at the intersection of the AIIS lateral wall and the iliac body. The screw should be inserted under the line between the midpoint of the AIIS and PSIS upper flat region to ensure accurate placement. The rate of cortical perforation can be significantly decreased under the guidance of the newly proposed canal.

## Data Availability

All the data was contained in the article.
